# P-2188. Hepatitis C Screening: Before and After Study of Hepatitis C Risk Score Alerts in CHORUS

**DOI:** 10.1093/ofid/ofae631.2342

**Published:** 2025-01-29

**Authors:** Douglas Dieterich, Rachel P Weber, Ricky K Hsu, Carl Millner, Jennifer S Fusco, Annie Son, Bruce Kreter, Andrew Stepp, Qin Ye, Gregory P Fusco

**Affiliations:** Mount Sinai Healthcare System, New York, New York; Epividian, Inc., Raleigh, North Carolina; AIDS Healthcare Foundation/ NYU School of Medicine, New York, New York; AIDS Healthcare Foundation, Los Angeles, California; Epividian, Inc., Raleigh, North Carolina; Gilead Sciences, Foster City, California; Gilead Sciences, Foster City, California; ZS Associates, Los Angeles, California; ZS Associates, Inc., Fort Myers, Florida; Epividian, Inc., Raleigh, North Carolina

## Abstract

**Background:**

Hepatitis C (HCV) screening rates remain low despite recommendations of one-time screening for asymptomatic adults and annual screening for people at higher risk of HCV acquisition. We sought to determine if alerts in CHORUS™, a clinical decision support system (CDSS), could improve the screening to diagnosis ratio.

**Figure:**
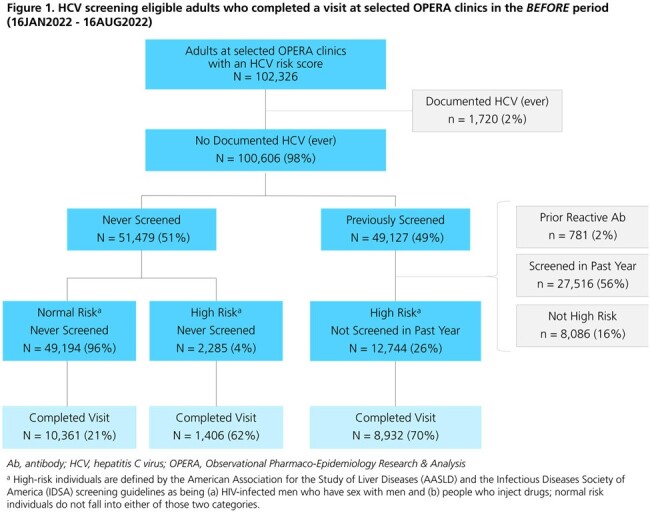

**Methods:**

A machine learning-based model calculated a percent chance of returning a positive HCV test for individuals eligible for HCV screening at selected OPERA^®^ clinics. Alerts providing the risk score and factors contributing to the score were disseminated to clinics via CHORUS ahead of their upcoming visits in the 16JAN2023-16AUG2023 period (with alerts) but not the 16JAN2022-16AUG2022 period (without alerts). Individuals were then followed through 17OCT of each period; HCV testing and diagnosis were described among individuals who completed a clinic visit.

**Figure:**
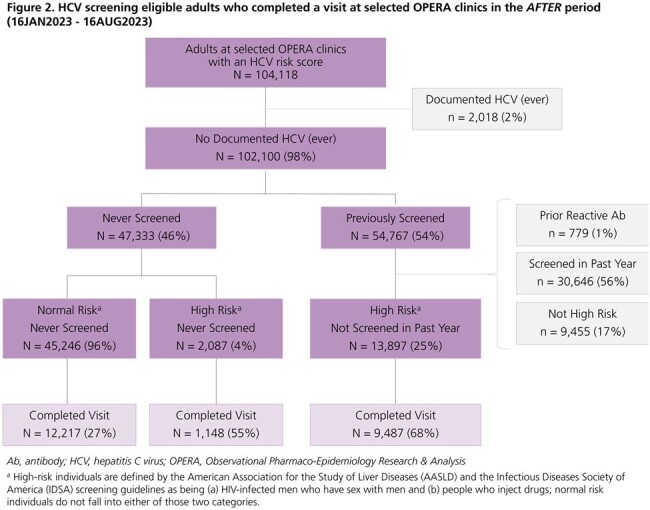

**Results:**

A total of 20,699 (*before* period) and 22,852 (*after* period) HCV screening-eligible individuals visited the clinics (Figures 1 & 2). Population characteristics were similar between study periods (Table 1). The proportions of individuals with a detectable HCV viral load (VL) among those who received any HCV test (antibody [Ab], VL, or genotype) over follow-up were 1% in both the *before* (36/4930) and *after* (40/5249) periods. However, the proportions of individuals receiving a VL test after a reactive Ab test and of those, the proportions with active HCV infection, were higher in the *after* period (94% & 56%) than in the *before* period (85% & 42%) (Figure 3).

**Figure:**
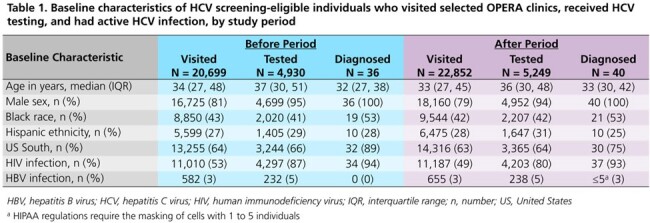

**Conclusion:**

In this *before* and *after* study, the proportion of screened individuals with active HCV infection was the same in both study periods, regardless of the alerts. Since HCV risk scores were disseminated for all 22,852 individuals ahead of their visits in the *after* period, alert-fatigue may have occurred. Additionally, providers may be cautious in following artificial intelligence-guided alerts. However, the proportion of VL tests with detectable virus was much higher after alerts, possibly suggesting heightened awareness of risk factors from the alerts or of HCV in general. As the HCV classification model is refined, future studies may consider alerting for only individuals with a high percent chance of infection.

**Figure:**
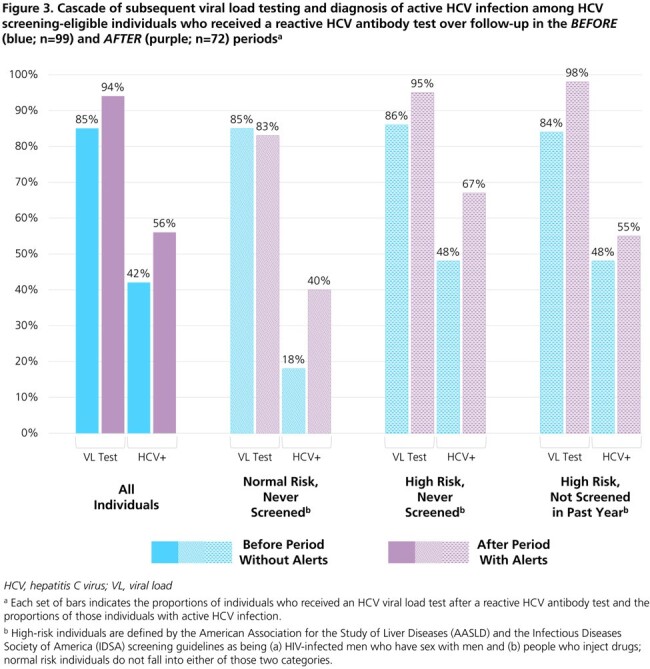

**Disclosures:**

Douglas Dieterich, MD, Gilead: Advisor/Consultant|Gilead: Honoraria Rachel P. Weber, PhD, EMD Serono: Research support to my employer|Gilead Sciences: Research support to my employer|Merck & Co.: Research support to my employer|TheraTechnologies: Research support to my employer|ViiV Healthcare: Research support to my employer Ricky K. Hsu, MD, EMD Serono: Advisor/Consultant|EMD Serono: Honoraria|Gilead: Grant/Research Support|Gilead: Honoraria|Merck: Honoraria|ViiV: Advisor/Consultant|ViiV: Grant/Research Support|ViiV: Honoraria Jennifer S. Fusco, BS, EMD Serono: Research support to my employer|Gilead Sciences: Research support to my employer|Merck & Co.: Research support to my employer|TheraTechnologies: Research support to my employer|ViiV Healthcare: Research support to my employer Annie Son, MBA, Gilead Sciences: Employee|Gilead Sciences: Stocks/Bonds (Public Company) Bruce Kreter, PharmD, Akero Therapeutics: Stocks/Bonds (Public Company)|Bristol-Myers Squibb: Stocks/Bonds (Public Company)|Eli Lilly: Stocks/Bonds (Public Company)|Gilead Sciences: Stocks/Bonds (Public Company)|Merck: Stocks/Bonds (Public Company)|Novo Nordisk: Stocks/Bonds (Public Company)|Pfizer: Stocks/Bonds (Public Company) Gregory P. Fusco, MD, MPH, EMD Serono: Research support to my employer|Gilead Sciences: Research support to my employer|Merck & Co.: Research support to my employer|TheraTechnologies: Research support to my employer|ViiV Healthcare: Research support to my employer

